# In silico characterization of bacterial chitinase: illuminating its relationship with archaeal and eukaryotic cousins

**DOI:** 10.1186/s43141-021-00121-6

**Published:** 2021-01-25

**Authors:** Bhramar Dutta, Jan Deska, Rajib Bandopadhyay, Salem Shamekh

**Affiliations:** 1 Juva Truffle Center, Huttulantie 1C, Juva, Finland; 2 Department of Botany, The University of Burdwan, Golapbag, Purba Bardhaman, West Bengal 713104, India; 3 Department of Chemistry and Materials Science, Aalto University, P.O. Box 11000 (Otakaari 1B), FI 00076 Aalto, Finland

**Keywords:** Chitinase, Phylogenetic relationships, Physical parameters, Structural and functional analysis

## Abstract

**Background:**

Chitin is one of the most abundant biopolymers on Earth, only trailing second after cellulose. The enzyme chitinase is responsible for the degradation of chitin. Chitinases are found to be produced by wide range of organisms ranging from archaea to higher plants. Though chitin is a major component of fungal cell walls and invertebrate exoskeletons, bacterial chitinase can be industrially generated at low cost, in facile downstream processes at high production rate. Microbial chitinases are more stable, active, and economically practicable compared to the plant- and animal-derived enzymes.

**Results:**

In the present study, computationally obtained results showed functional characteristics of chitinase with particular emphasis on bacterial chitinase which is fulfilling all the required qualities needed for commercial production. Sixty-two chitinase sequences from four different groups of organisms were collected from the RCSB Protein Data Bank. Considering one suitable exemplary sequence from each group is being compared with others. Primary, secondary, and tertiary structures are determined by in silico models. Different physical parameters, viz., pI, molecular weight, instability index, aliphatic index, GRAVY, and presence of functional motifs, are determined, and a phylogenetic tree has been constructed to elucidate relationships with other groups of organisms.

**Conclusions:**

This study provides novel insights into distribution of chitinase among four groups and their characterization. The results represent valuable information toward bacterial chitinase in terms of the catalytic properties and structural features, can be exploited to produce a range of chitin-derived products.

**Supplementary Information:**

The online version contains supplementary material available at 10.1186/s43141-021-00121-6.

## Background

Chitinases (EC 3.2.1.14) are glycosyl hydrolases that catalyze the hydrolytic degradation of chitin. Chitin represents the second most abundant carbohydrate after cellulose, occurring as natural insoluble biopolymer featuring linear β-1,4-linkages between *N*-acetyl d-glucosamine (GlcNAc). It is extensively distributed in nature in different forms and constitutes, among other things, an essential component of the shells of crustaceans and exoskeletons of insects, as well as the key component in cell walls of a variety of fungi.

*Aspergillus*, *Trichoderma*, etc. are common fungal strains known to produce chitinase [1, 2]. Besides the fungal sources, many bacterial genera including *Serratia* sp., *Streptomyces* sp., and *Bacillus cereus* produce high levels of chitinolytic enzymes [3]. Also, some archaea like *Pyrococcus furiosus* [4] or *Thermococcus kodakarensis* [5] can produce chitinase under hyperthermophilic conditions. Furthermore, just recently, a new family of chitinase from *Thermococcus chitonophagus* has been reported by Horiuchi et al. [6] which contains two additional chitin-binding domains along with catalytic domains.

Those chitinases are found to be active in a broad temperature range with a good pH tolerance. Whereas endochitinase from mesophilic actinomycetes *Streptomyces violaceusniger* can tolerate temperatures up to 28 °C [7], S*treptomyces thermoviolaceus* produces thermostable chitinase withstanding 80 °C at pH 8–10 [8], and the mesophilic bacterium *Aeromonas hydrophila* provides chitinolytic proteins performing at 37 °C and pH 8.0 [9]. On the opposite side of extremophilic behavior, the psychrophilic bacterium, *Arthrobacter* sp. from the Antarctic sea sediment, is reported by Lonhienne et al. to secrete chitinase at 4 °C [10], while *Stenotrophomonas maltophilia* [11] shows an acidic pH optimum range of 4.5 to 5.0 for production of chitinase enzymes.

The diverse role of plant chitinase varies from the evolutionary oldest moss to highly evolved monocot plants. They are not only involved in the defense response but also implicated in symbiotic associations, organ development, and resistance to abiotic stresses. *Bryum coronatum* is a moss that produces chitinase-A as safeguard from fungal pathogen [12]. Class III chitinase from *Vitis vinifera* [13] including fern genera *Pteris ryukyuensis* [14] features a specific two lysine motif for enhancing resistance against fungi. Moreover, chitinase-A from *Cycas revoluta* expresses additional transglycosylation activity which is unique in the plant kingdom [15]. The saffron chitinase from *Crocus sativus* [16] promotes the jasmonic acid signal pathway as defense activation mechanism.

Chitinase enzymes have a broad range of applications such as preparation of pharmaceutical products, production of single-cell proteins [17, 18], isolation of protoplasts from fungi [19], biomarker for diagnosis of cancer [20] as well as controlling malaria transmission.

According to Oerke et al. [21], around 35% of crop yields are lost due to diseases in the field and 15% covers the post-harvest loss. In this preventive aspect, microbial chitinases offers the opportunity for cell wall degradation of many pests and pathogens, thereby unveiling antibacterial, antifungal, insecticidal, and nematocidal activity [22].

A multidisciplinary approach is necessary for the successful integration of chitinase in industrial scale applications as well as multi-fold product yield. An efficient enzyme production depends on desired physical, chemical, and mechanical properties of the biocatalysts. Herein, computational design plays an appreciable role to understand the overall properties of bacterial chitinase in comparison with archaeal, fungal, and plant-derived analogs. Therefore, a co-ordinated study following the recent trends of genetic engineering would be helpful to use chitinolytic microorganisms in rendering plant defense.

## Methods

### Retrieval of the sequences from RCSB PDB

A total of 62 different chitinase sequences of archaeal, bacterial, fungal, and plant origin have been retrieved from the RCSB protein data bank (http://www.rcsb.org) on 4 October 2018.

### Determination of physical parameters

Different physiochemical characteristics of chitinase enzymes were analyzed by means of using the ExPASy–ProtParam tool (http://web.expasy.org/protparam) [23]. Computational approaches have been employed covering parameters such as molecular weight, theoretical pI value, instability index, aliphatic index, and grand average of hydrophobicity (GRAVY).

### Analysis of primary structure

The core amino acids that form the primary structures of the proteins are extracted and listed by using the ExPASy–ProtParam tool.

MEME Suite 5.0.2 was utilized to detect any signature sequence of chitinase enzyme present in all the evaluated organisms (http://www.meme-suite.org) [24]. MEME-ChIP was performed using classic mode of motif discovery. Default width of MEME motif was set to 3 and 20 as minimum and maximum motif respectively.

### Analysis of secondary structure

Analysis of the secondary structure by SOPMA has been performed (https://npsa-prabi.ibcp.fr), according to Geourjon and Deleage [25].The PDBsum tool provides the pictorial overviews of the macromolecular structures (http://www.ebi.ac.uk/) showing the possible arrangements of long α-helices and large β-sheets in this resolution. Ramachandran plots were generated by the PROCHECK tool (http://servicesn.mbi.ucla.edu/PROCHECK/) [26–28].

### Analysis of tertiary structure

The overall quality of the constructed 2D model has been evaluated with ERRAT value (services.mbi.ucla.edu/ERRAT) [29]. ProSA-web was exploited to assess the *Z* score and energy plots (https://prosa.services.came.sbg.ac.at/prosa.php) [30]. Validation of the homology modeling of protein 3D structures was performed on a SWISS MODEL web-server (https://swissmodel.expasy.org/interactive/6Dk7as/models/) [31–33]. QMEAN comprised three different models (QMEAN local quality, DisCo, Brane) of assessment to understand the geometrical features of the protein model (https://swissmodel.expasy.org/qmean/). Finally, QMEAN 4 was used to fit cumulative QMEAN value in global scale at a range of 0 to 1 (https://swissmodel.expasy.org/qmean/).

### Functional analysis

The protein family shares common evolutionary pathways among other groups of organisms. Contrarily, GO (gene ontology) defines the classes used to describe the gene function, from molecular activities of gene product to large biological pathways. To understand both these purposes, the InterProScan server (www.ebi.ac.uk/interpro/search/sequence-search) was used.

The interacting proteins involved in chitinase enzymes were displayed by STRING server (https://string-db.org/) [34].

The SBASE tool (http://pongor.itk.ppke.hu/protein/sbase.html) facilitated the detection of protein domain sequences representing various structural and functional segments of proteins.

The knowledge of protein membrane topography is very crucial to assess how they react with outer side biomolecules. The TMHMM tool was used (http://www.cbs.dtu.dk/services/TMHMM/) to predict whether the protein is membrane-spanning or extracellular in nature.

Subcellular localization of the protein was confirmed by the mGOASVM tool (http://bioinfo.eie.polyu.edu.hk/mGoaSvmServer/mGOASVM.html).

In proteomics studies, the cutting of proteins and polypeptides to smaller peptides is essential for mass spectrometric analysis. The Peptide Cutter tool predicts computational substrate cleavage site of a protein by endopeptidase or chemical treatment (web.expasy.org/peptide_cutter/).

### Construction of phylogenetic tree

Phylogenetic analysis was performed using the MEGA 10.0.5 software [35] with all of the 62 sequences. Alignment of amino acid sequences was achieved by CLUSTAL W. Maximum likelihood method was used for generating tree with a Poisson correction model for multiple amino acid substitution with 1000 random bootstrap replicates. The trees were optimized with heuristic nearest neighbor interchange (NNI) method.

## Results

### Sequence retrieval from RCSB PDB

Sixty-two different chitinase enzyme sequences from four different groups of organisms have been retrieved. Both FASTA and PDB formats were recorded as input to perform computational studies. Here, we were considering one organism as candidate(s) from each four groups based on the specificity.

### Comparison of physiochemical parameters

A comparison of physiochemical properties with the four groups of organisms is presented in Table 1. Stability of all sequences has been studied by analyzing the values for the instability index, the aliphatic index, and the grand average of hydropathicity (GRAVY) index.

**Table 1:** Comparison of physiochemical parameters of chitinase derived from four different organisms computed using the ExPASy ProtParam tool

PDB ID	Organism	Amino acid	Molecular weight	pI	Instability index	Aliphatic index	GRAVY (-)
**2DSK**	Archaea	311	334,733.41	4.75	18.63	84.63	− 0.137
**1EDQ**	Bacteria	541	58,712.04	5.67	15.39	74.31	− 0.322
**2Y8V**	Fungi	310	32,472.07	5.35	46.93	87.72	− 0.251
**3AQU**	Plants	356	38,642.03	8.84	32.62	70.22	− 0.193

Proteins whose instability index are lower than 40 are predicted by Guruprasad et al. [36] to be stable while levels above 40 indicate unstable sequences. The evaluated bacterial chitinase here (with an instability index of 15.39) appears as stable protein sequence in this analysis. Fungi and higher plants are predicted to produce unstable chitinase with relatively high instability indices compared to bacteria.

The aliphatic index measures the relative volume occupied by aliphatic side chains of a given protein. An increase in the aliphatic index increases the thermostability of globular proteins, and consequently, a high aliphatic index of 74.31 accounts for a good temperature stability of the bacterial protein [37, 38].

The isoelectric point (pI) expresses the pH at which an amino acid, peptide, or protein does not migrate in an electric field. The ProtParam analysis displayed the theoretical pI value for the bacterial chitinase as 5.67 indicating as negative charge of the protein, predicting it to be acidic in nature. In contrast, plant chitinases are found to be preferably basic in nature (pI value 8.84).

### Primary structure determination

**Fig. 1:**
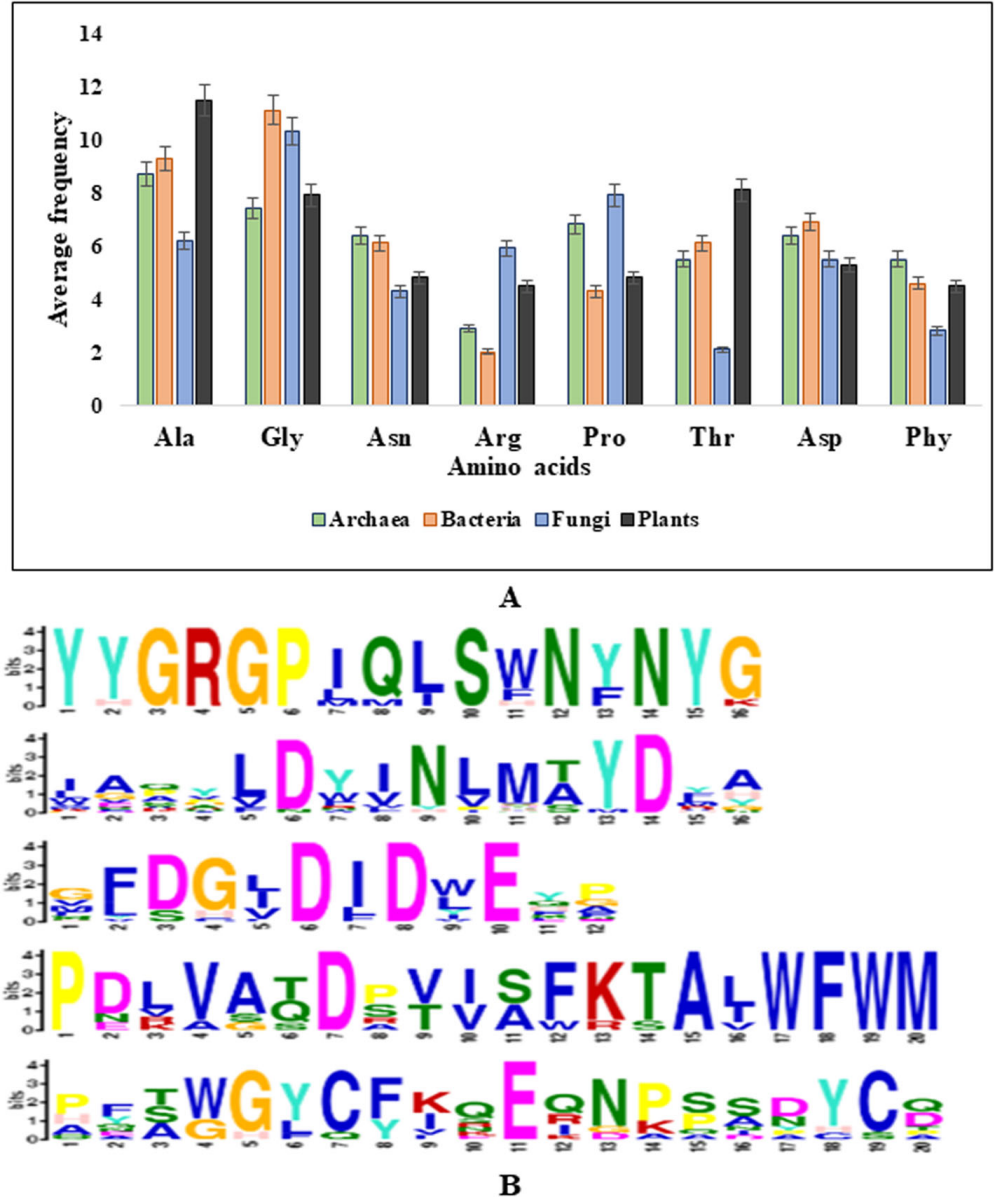
Distribution of amino acid and conserved residues of amino acids existing in the primary structure. **a**. Graphical representation of the contributing dominant amino acids involved in the primary structure of chitinase protein from different organisms. **b** Conserved sequence motifs elicited by MEME suite present in all the 62 sequences

Proteins differ from one another by their structures, primarily in their sequences of amino acids. The abundance of certain amino acids determines different structural organizational elements of proteins. Eight prevalent amino acids are found in the structure of bacterial chitinases. The average percentage is displayed as follows: alanine (9.3%), glycine (11.1%), asparagine (6.1%), arginine (2.0%), proline (4.3%), threonine (6.1%), aspartic acid (6.9%), phenylalanine (4.6%). Figure 1a shows the comparative percentage of amino acids present in archaea, fungi, and plants.

The MEME suite tool found significant consensus motifs (*E* value ≤ 0.05). Five functional motifs are identified from all the sequences represented in Fig. 1b.

### Secondary structure determination

The understanding of the secondary structures is of paramount importance as this configuration elucidates the reactive nature of proteins.

**Fig. 2:**
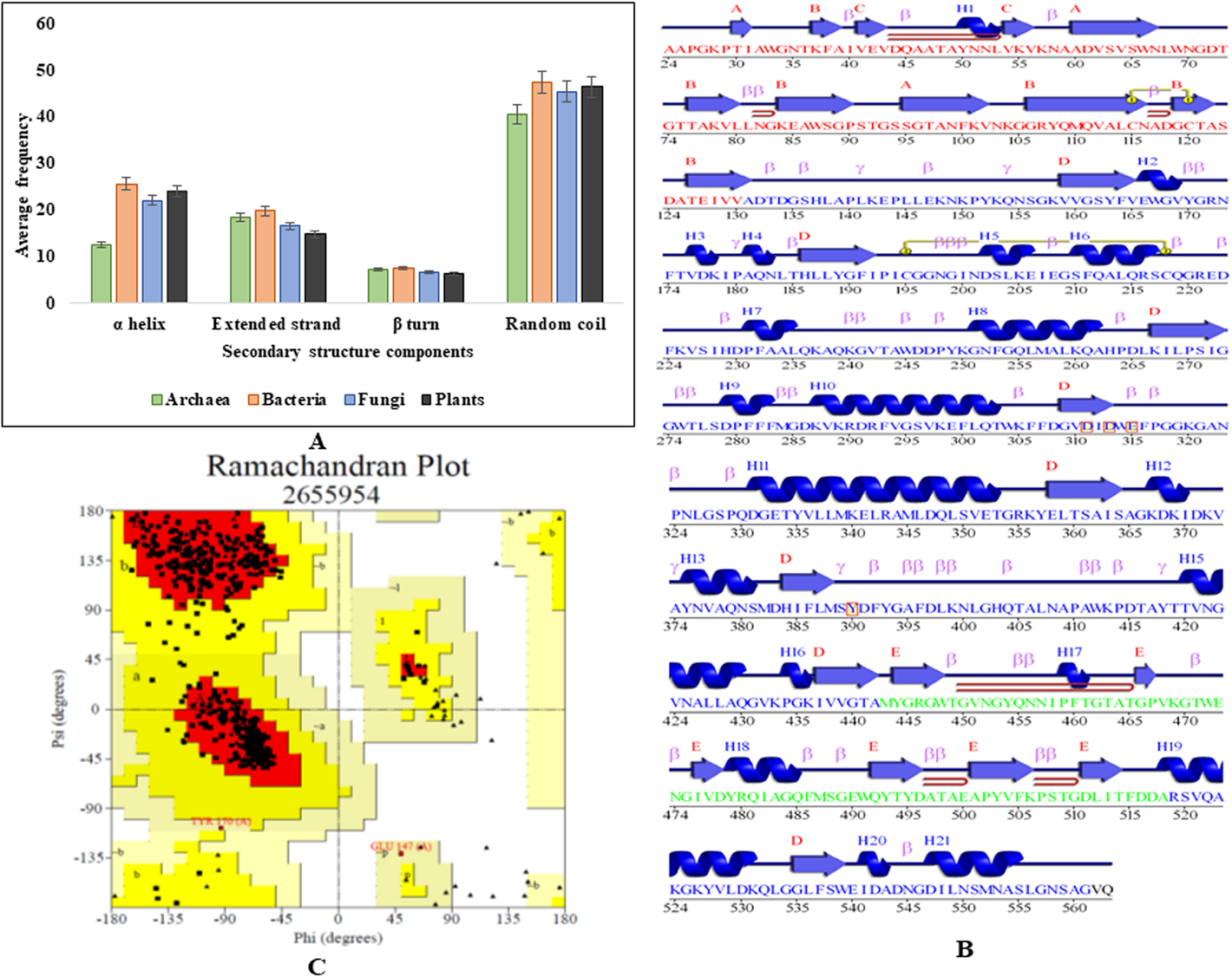
Assessment of secondary structure of chitinase. **a** Bar graph representing the distribution of three types of secondary structures elements present in chitinase. **b** Schematic wiring diagram of bacterial chitinase (PDB ID 1EDQ). **c** Ramachandran plot of the bacterial chitinase (PDB ID 1EDQ) showing the distribution of amino acids phi/psi angles

The SOPMA tool revealed that chitinase enzymes are dominated by random coils which pointed toward evolutionary conserveness of the proteins. In addition, random coils are often described as regions where the folded chain acts more flexibly and dynamically than other secondary conformational structures [39]. According to Shortle [40], no sidechain-sidechain interaction occurs along the polypeptide chain, and the connecting bridges between beta strand and alpha helix is supposed to be the most conserved region of random coils. Figure 2a shows comparative percentage of random coils, α helices, extended strands, and β turns within all four groups of organisms.

PDBSum shows that the bacterial chitinase contained three domains. Domain 1 mainly features β-sandwich, domain 2 α-β-barrel, and domain 3 consists of an α-β-roll.

Figure 2b shows a β-hairpin in between residues 45 and 55, as well as small hairpins between 115 and 120, 450 and 465, 495 and 500, and between 504 and 510. Residues 195/200 as well as 204/215 are connected with disulfide bridges.

The PROCHECK tool was exploited to generate the Ramachandran plot (Phi/Psi) of proteins. Figure 2c shows the 90.8% residues falling in most favored region denoted as core by the red color, indicating a good quality of model. The brown-colored region is covering 8.8% of residues in additionally allowed region. The dark yellow-colored region contains 0.4% residues in generously allowed regions. Although four groups of sequences showed approximately 90% of residues falling in favored regions in Table 2, there are no residues under the white-colored disallowed region in bacterial protein. Residues tend to fall in disallowed regions because of steric hindrance or clashes between atoms. Active amino acids in disallowed region indicate the declination of stability of protein [41].

**Table 2:** Comparison of distribution of amino acids in the Ramachandran plot provided by the PROCHECK program

PDB ID	Organism	Residues in favored region (%)	Residues in additional allowed region (%)	Residues in generously allowed region (%)	Residues in disallowed region (%)
2DSK	Archaea	90.8	8.4	0.4	0.4
1EDQ	Bacteria	90.8	8.8	0.4	–
2Y8V	Fungi	89.5	10.0	–	0.4
3AQU	Plants	90.7	9.0	–	0.3

### Tertiary structure determination

The protein molecule bends and twists in such a fashion to achieve maximum stability on lowest energy state resulting in a defined overall three-dimensional shape. The selected bacterial chitinase model was evaluated using the ERRAT server. The error values that fall below 95% are measured in rejection limit [38]. In Table 3, ERRAT showed an overall quality factor of 95.085, a result expected for crystallographic models with resolutions > 2.5 Å. Although the overall ERRAT quality factor value lies above 95% (except archaea) in bacteria, fungi, and higher plants, further model validation tools suggest that these are not as reliable with high confidence as bacteria.

**Table 3:** Comparison of ERRAT quality factors for the assessment of three-dimensional structures

PDB ID	Organism	ERRAT quality factor (%)
2DSK	Archaea	93.493
1EDQ	Bacteria	95.085
2Y8V	Fungi	95.221
3AQU	Plants	97.917

**Fig. 3:**
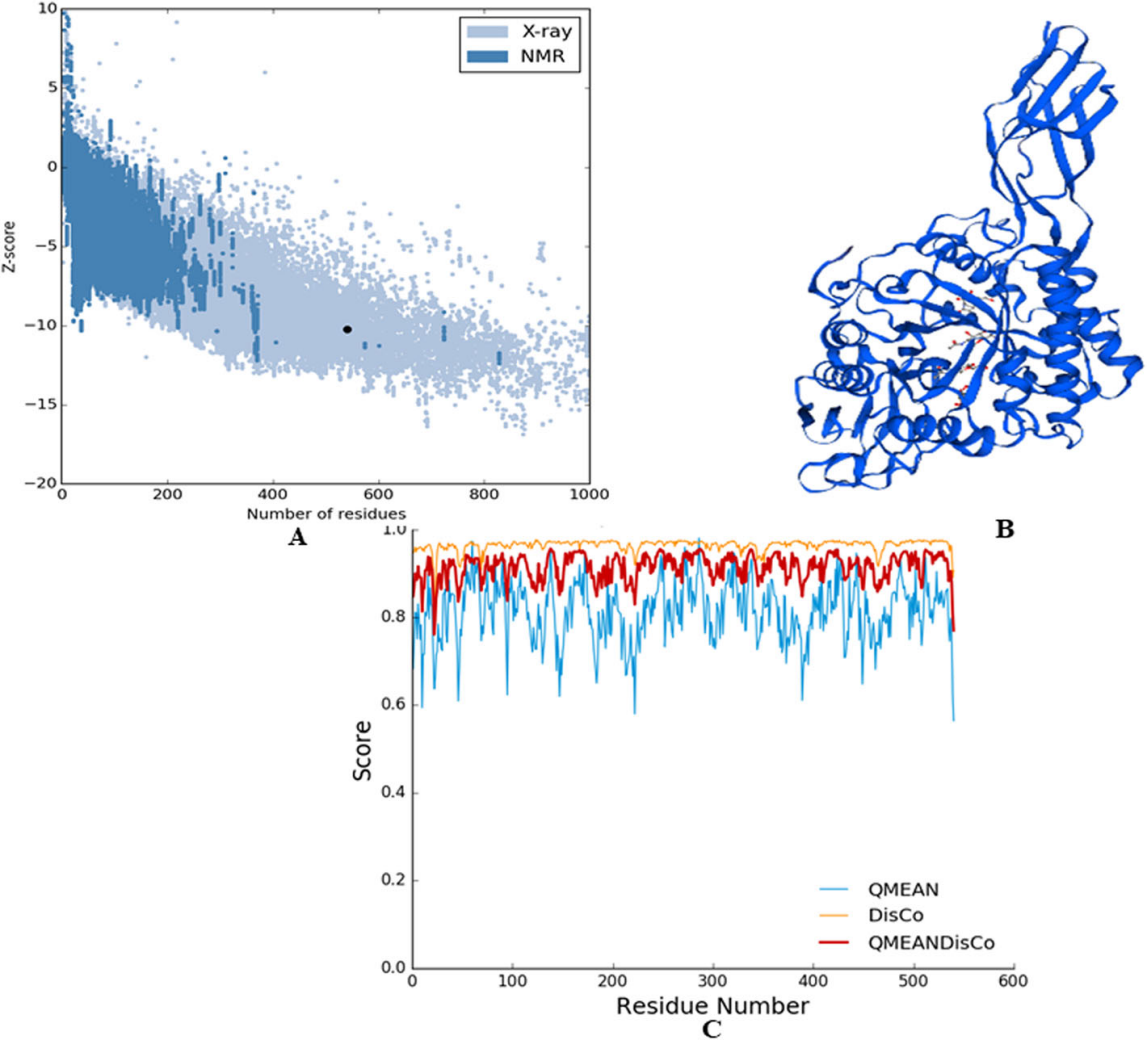
Visualization of modeled tertiary structure of chitinase. **a** *Z* score value of bacterial chitinase (PDB ID 1EDQ) generated by ProSA server for 3D model validation. **b** 3D structure of bacterial chitinase (PDB ID 1EDQ) generated by SWISS-MODEL Workspace. **c** QMEAN DisCo Score of bacterial chitinase (PDB ID 1EDQ) indicating predicted 3D model has X-ray diffraction quality

ERRAT value was further normalized with the protein size and quantitatively assessed by highresolution X-ray crystallography. ProSA generated Z score for bacterial sequence (PDB ID:1EDQ) is −10.23. The score fits well within the range indicating a highly stable structure. A plot(Fig. 3a) showing normalized Z score on the Y axis versus number of residues on the X axis found to be well in tolerable limits. SWISS MODEL Workspace visualizes the final 3D model. A total of 26 templates were found for the bacterial chitinase (PDB ID: 1EDQ). The best fit model generated by model-template alignment in Fig. 3b was based on the top five templates. The templates were selected for model building based upon the highest quality of sequence identity, best *E* value, and maximum number of query sequence covered. Four *N*-acetyl d-glucosamine residues bind as ligand molecules with protein. The generated model was estimated by assessing cumulative QMEAN (QMEAN local quality, DisCo, Brane) score and is displayed in Fig. 3c. Larger QMEAN scores indicate better models whereas negative scores refer to unstable models [32]. Figure 3c shows that QMEAN scored 0.65 which provides an estimation of the degree of nativeness. 0.65 is very well fitted within the expected range as the standard deviation is less than 1 from the mean score, very similar to related results by Benkert et al. [32]. Furthermore, QMEAN4 is a reliability score based on four linear combinations—local geometry, distance-dependent interaction, agreement of the predicted secondary structure and solvent accessibility, solvation potential calculation. The global model reliability estimation (GMQE) for QMEAN4 ranged between 0 and 1 [42]. Comparing with other groups of organisms from Table 4, only bacterial proteins fit under the GMQE scale.

**Table 4:** Comparison of QMEAN4 values for the assessment of three-dimensional structures

PDB ID	Organism	QMEAN4 value
2DSK	Archaea	1.27
1EDQ	Bacteria	0.78
2Y8V	Fungi	1.88
3AQU	Plants	− 0.13

### Analysis of protein function

InterPro Scan provides information regarding the functional protein families. In all the four organisms, chitinase belongs to the glycoside hydrolase superfamily. Figure S(A) shows bacterial chitinase belonging more specifically to glycoside family 18 featuring both a catalytic domain and chitin-binding domain. For the overall knowledge about protein functions, it is necessary to understand protein-protein interaction. STRING database infer the protein association with other proteins regulated by co-operative binding [43]. An illustration from Fig. S(B) interlinks ten interacting chitin-binding domains containing proteins within the glycoside hydrolase family. Spro_2725 is our query protein and shows interplay with eight Spro proteins and one nagK and nagZ proteins.

Two proteins, *N*-acetyl d-glucosamine (nagK) and β-hexosaminidase (nagZ), are made up of 306 amino acids and 339 amino acid residues respectively. nagK is involved in phosphorylation of GlcNAc to produce GlcNAc-6-P supporting the glycosylation signaling pathways. Muropeptides are hydrolyzed to 1,6-anhydromurNAc by the nagZ protein in the cytoplasm. Both of the proteins are associated with amino sugar and peptide recycling. Furthermore, MurNAc-6-phosphate was converted to GlcNAc-6-P by an etherase enzyme. GlcNAc-6-P is then deacetylated and either enters glycolysis or remains as precursor of chitin-like tertiary compounds for murein biosynthesis.

The domain included in SBASE represents bacterial chitinase composed of 540 amino acids. Figure S(C) showed one core domain and one ligand-binding domain.

The TMHMM result depicts that there is no transmembrane portion in either of the four sequences. It clearly indicates the extracellular production nature of chitinase enzymes [44].

The mGOASVM results confirmed the subcellular localization of chitinase in the cytoplasm.

**Fig. 4:**
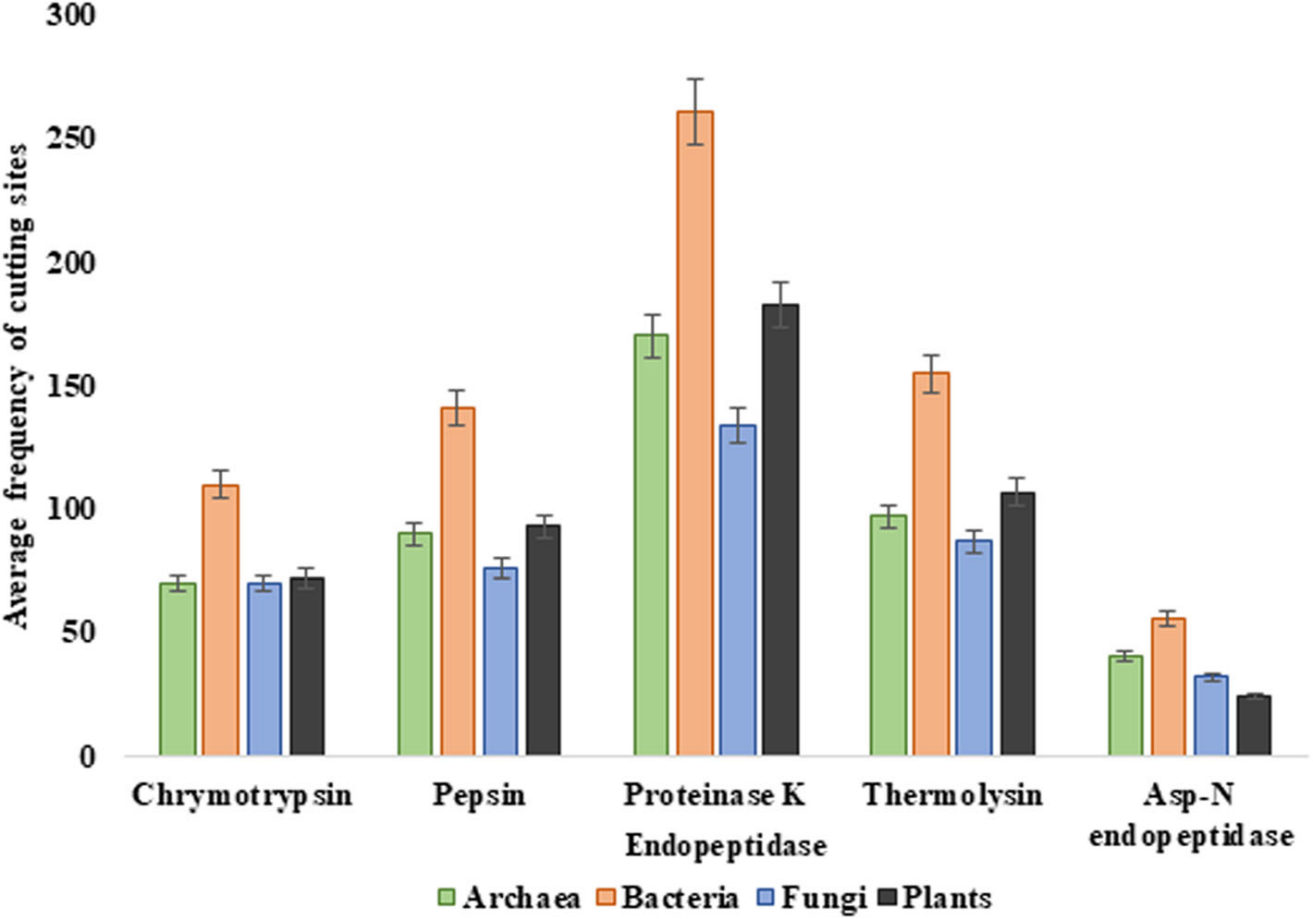
Column graph showing average number of cleavage sites for the enzyme chitinase as identified through the peptide cutter tool

Proteolytic cutting of enzymes has utmost importance. A comparative study among the four groups of organisms with five different types of endopeptidases, viz., chymotrypsin, pepsin, proteinase K, thermolysin, and Asp-N terminal endopeptidase, having highest cutting sites have been given in Fig. 4.

### Phylogeny study of chitinase

**Fig. 5:**
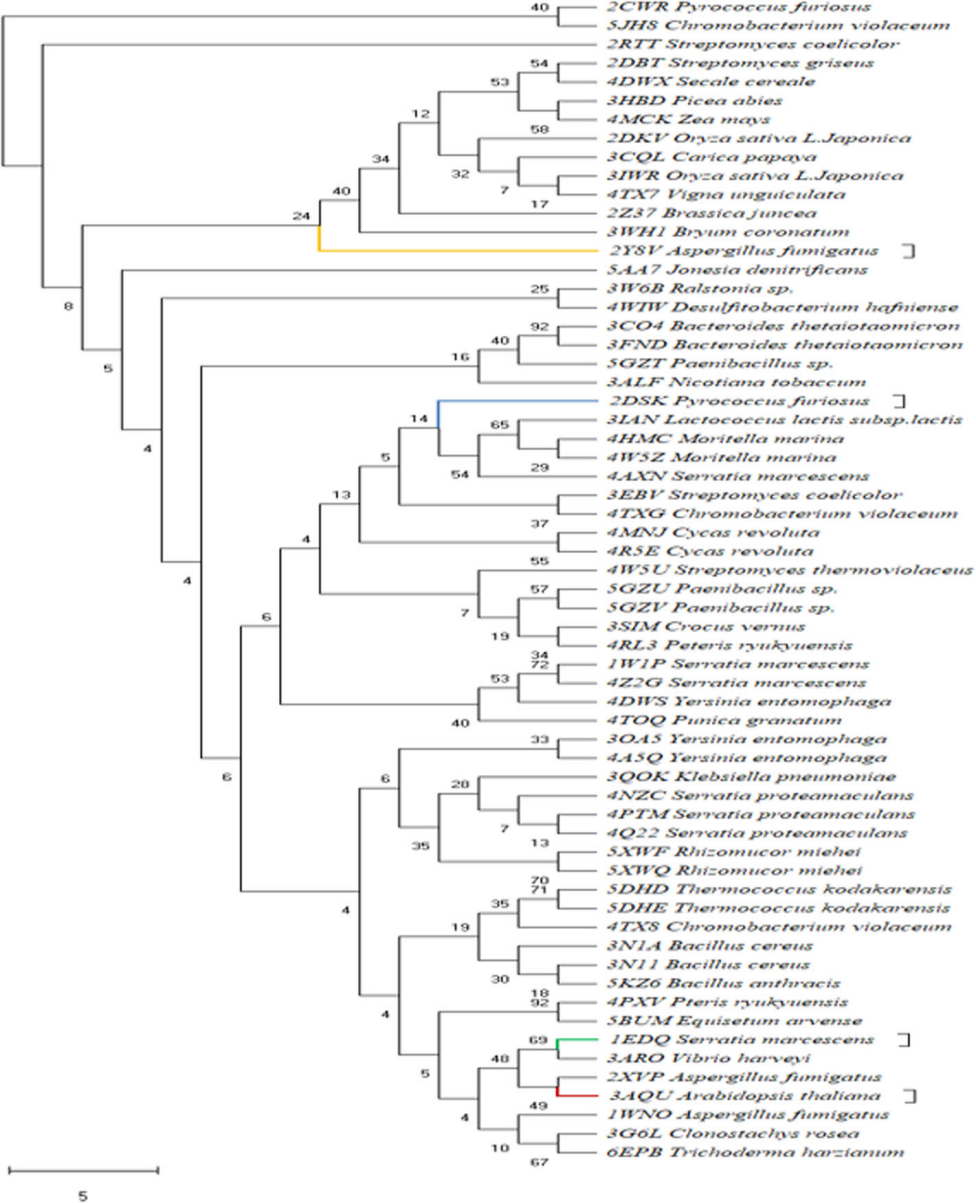
Phylogenetic tree of all the 62 chitinase sequences constructed using maximum likelihood method in the MEGA 10.0.5 software. Bootstrap values are depicted at the nodes

Sixty-two sequences were found to be distributed in all fourteen clades. Figure 5 shows that each clade was divided into branches, and the branches are subdivided further into subbranches. The fungal sequence 2Y8V, from *Aspergillus fumigates*, was placed in a separate branch line. In the second clade, the archaeal sequence 2DSK from *Pyrococcus furiosus* (PDB ID 2DSK) got its position at same branch length with bacteria. 1EDQ from *Serratia marcescens* situated in a different clade with other bacteria. The plant sequence 3AQU from *Arabidopsis thaliana* shared the position with fungi, shedding some light that they are evolutionary closely related.

Fossil relics tell an evolutionary story of chitinase. In fossil-calibrated dating studies, molecular clock-measured time ensured that multiple clades of bacteria gathered chitinase by horizontal gene transfer during late Neoproterozoic-Cambrian transition to the early Paleozoic [45].

The phylogenetic study characterizes GH chitinase family 18 clusters A and B that is widely distributed in three domains of life, whereas family 19 is predominant in plants and some Actinobacteria like *Streptomyces* sp. [46]. Extra copies of genes are inserted by duplication and are supposed to diverge in plants. The GH18 cluster C includes bacterial and Archaean representatives.

The evolutionary lineage depicts that fungi colonized land during Cambrian, earlier than arthropods in Ordovician. Later, micro fungus-feeding insects, beetles, depend on two-way interaction between the two chitin-driven organisms.

## Discussion

From the current study, a definable similarity has been observed among four groups of organisms as well as structural and functional alterations obtained from the analysis. In our work, the average results of a given parameter are being used as taking all the sequences would have made the analysis too exhaustive. One well-studied exemplary member from each group, i.e., the archaeal model *Pyrococcus furiosus* (2DSK), the bacterial model *Serratia marcescens* (1EDQ), the fungal model *Aspergillus fumigates* (2Y8V), and the plant model *Arabidopsis thaliana* (3AQU), suffices to characterize different empirical results. The features include that they are able to perform all the computational tools being used in this study and the model connectivity correlated with the rest of the member in that group. Very high value of aliphatic indices was found in archaea (84.63) and fungi (87.72) whereas higher plants feature significantly lower aliphatic values (70.22). Thus, the order of great thermal stability plants < bacteria < archaea < fungi suggests that thermophilic fungi are close cousin to archaea having a high aliphatic value. The moderate aliphatic range in bacteria suggests the ancestry of chitinase in higher plants and fungi originated form bacteria. This hypothesis is based on the earlier studies by Gruen et al. that showed chitinase gene from bacteria radiated toward plant and fungal lineage [45]. Onaga and Taira [14] identified that the LysM domains of plant class IIIb chitinase shared moderate level of homology to fungal (*Aspergillus fumigatus)* LysM domain proteins. The resemblance shown by plant and fungal chitinases revealed that the catalytic domains are similar in all of these.

Very high pI values in plants are relatable to the modified five kingdom system carried out by Margulis and Schwartz [47], where different entities belong to the prokaryote and four eukaryotic kingdoms—the Protocista, the Fungi, the Plantae, and the Animalia. This is regarded as primarily acidic chitinases arise in bacteria, and gradually basic nature of chitinases are found in plants.

On the other hand, the three domain classifications, a category above Kingdom, proposed by Woese [48], nucleotide sequences of ribosomal rRNA act as an evolutionary chronometer. It became obvious from our computed physiochemical analysis that there are several differences between archaea and bacteria although having prokaryotic origin. With all the characteristics studied, it has been realized that these bacterial chitinase are more closely related to the eukaryotes, especially plants.

A negative GRAVY value of − 0.322 suggests an overall non-polar and hydrophobic nature of the evaluated bacterial chitinase. Similar observations of Edbeib et al. [49] suggested that negative GRAVY values convey low hydrophobicity of proteins; the more negative value tends to improve interaction with water molecules. The GRAVY value indicates that chitinases evolved first in bacteria, and then diverged to fungi and plants. The amino acid composition depicted in Fig. 1a shows that alanine, glycine, and proline as hydrophobic amino acids tend to be in side chains, buried within the hydrophobic core of the protein, or within the lipid portion of the membrane. Arginine, threonine, and asparagine being the hydrophilic amino acids have a tendency to interact in the aqueous environment due to polarity and found on the exterior surface. Glycine with good percentage in the sequences gives high flexibility to the polypeptide chain and provides rigidity to the structure. Its presence in the surface provides a particular shape at these locations. By performing motif search, it has been observed that chitinase-specific domains are present in all organisms but in varied position. Secondary structural features were predicted by the SOPMA tool and indicate a high amount of random coil. Proline which is prominent in chitinases has the property to provide conformational rigidity and might be responsible for high content of random coiled structures. Rose [50] showed that coils contain more repetitive structure. The *Z* score signifies that the predicted tertiary structure is stable. Annotation with InterPro entries with GO (gene ontology) terms enabled to interpret biosynthetic pathway of chitinase derived from carbohydrate metabolism. Funkhouser and Aronson [51] addressed that the GH 18 multiprotein family of chitinases evolutionarily radiated from archaea to eukaryotes through gene duplication, loss, and selection process .The phylogenetic tree revealed that all the chitinase sequences were originated from archaea and diverged into subgroups during time and course of evolution. Above all, the presence of clusters for archaea and bacteria along with fungi and plants confirms the molecular level changes among the species during evolution.

## Conclusion

The present study findings strongly affirmed that the bacterial chitinase is thermostable, hydrophobic in nature with less occurrence of thermolabile residues. Due to non-reliability of some scores for other groups except bacterial enzyme, structural modeling was performed to predict the 3D structure which is stable and kinetically accessible. The chitinase sequences from archaea, bacteria, fungi, and plants have fundamental functional relationship, as they have motif identity. Plant model sequence shared same branch length with fungal sequence in the phylogenetic tree. Also, extreme high and low aliphatic indices of fungi and plants respectively throw some light on their close relationship. Bacteria having moderate aliphatic value and sharing the branch length with other bacteria provides a rational framework of chitinase among these four groups. In the future, robust computational methods combined with advance strategies will provide highly efficient microbe-based de novo industrial chitinase. Here, chitinolytic microorganisms have the potential as promising replacements for the more harmful practices of applying insecticidal and antifungal chemicals. From the present in silico study of chitinase, it is assured that bacteria could be used as a potential source of chitinase production posing an attractive alternative to commonly exploited fungal sources.

## Supplementary Information

**Additional file 1: Supplementary Figure 1.** Evaluation of functional annotations of chitinase. **Fig. S(A)**. InterPro Scan server showing (PDB ID 1EDQ) predicted protein family relationship, domains and sites of chitinase. **Fig. S(B)**. Protein-protein interacting partners of chitinase found through STRING database (PDB ID 4NZC). **Fig. S(C)**. Structural domains of bacterial chitinase (PDB ID 1EDQ) predicted by SBASE tool.

## Data Availability

Supplementary data associated with this article are available in the online version.

## Abbreviations

PDB: Protein Data Bank
pI: Isoelectric point
RCSB: Research Collaboratory for Structural Bioinformatics
GRAVY: Grand average of hydrophobicity
QMEAN: Qualitative Model Energy Analysis
GMQE: Global Model Quality Estimation
GO: Gene Ontology
MEGA: Molecular Evolutionary Genetics Analysis
MEME: Multiple Em for Motif Elicitation
GH: Glycoside hydrolase
GlcNAc: *N*-acetyl d-glucosamine
