# Supplementary figures and images for: In silico characterization of bacterial chitinase: illuminating its relationship with archaeal and eukaryotic cousins
Source: J Genet Eng Biotechnol. 2021 Jan 25;19:19. doi: 10.1186/s43141-021-00121-6 (PMC7835276; doi:10.1186/s43141-021-00121-6)

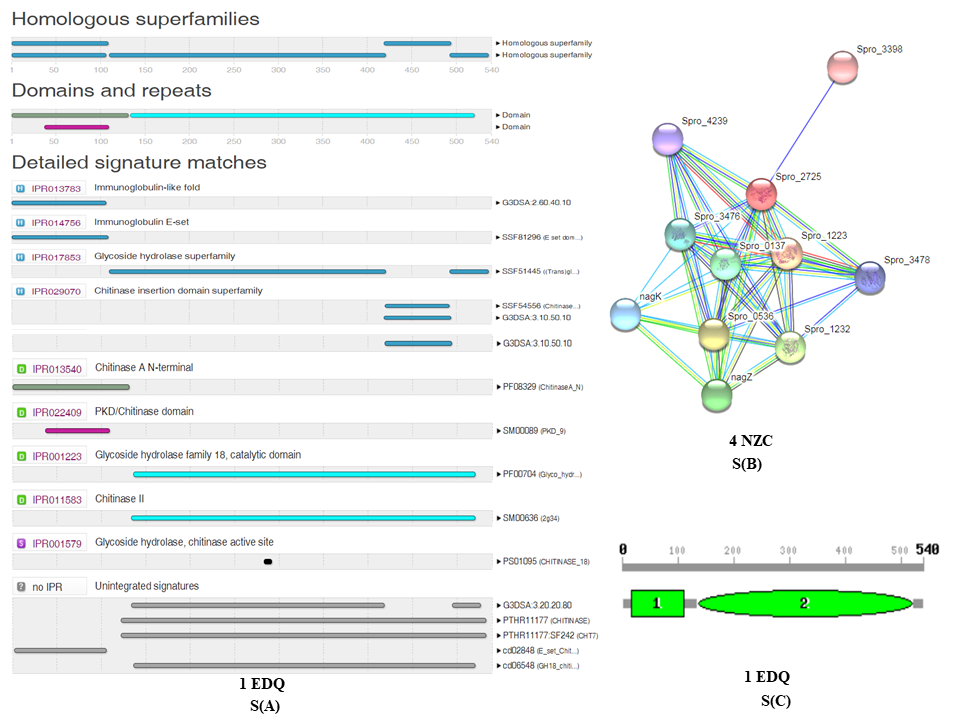

Supplement: Supplementary file 1 — Additional file 1: Supplementary Figure 1. Evaluation of functional annotations of chitinase. Fig. S(A). InterPro Scan server showing (PDB ID 1EDQ) predicted protein family relationship, domains and sites of chitinase. Fig. S(B). Protein-protein interacting partners of chitinase found through STRING database (PDB ID 4NZC). Fig. S(C). Structural domains of bacterial chitinase (PDB ID 1EDQ) predicted by SBASE tool. [file 43141_2021_121_MOESM1_ESM.tif]
